# Miniature planar telescopes for efficient, wide-angle, high-precision beam steering

**DOI:** 10.1038/s41377-021-00576-9

**Published:** 2021-06-28

**Authors:** Ziqian He, Kun Yin, Shin-Tson Wu

**Affiliations:** College of Optics and Photonics, University of Central Florida, Orlando, FL 32816 USA

**Keywords:** Adaptive optics, Micro-optics

## Abstract

Non-mechanical beam steerers with lightweight, compact, high-efficiency, high-precision, and/or large-angle are pivotal for light detection and ranging (LiDAR) of autonomous vehicles, eye-tracking for near-eye displays, microscopy, optical tweezers, and high-precision three-dimensional (3D) printing. However, even the most matured optical phased array can only provide quasi-continuous, efficient beam steering within a small angle range. A telescope module with an angle magnification function can be coupled to enlarge the steering range or precision. But obtaining a compact, low-cost, lightweight, high-quality telescope module with conventional optics remains challenging. Patterned liquid crystal-based planar optical elements offer great design freedom for manipulating the phase profile of light in 2D space. Owing to the advantages of high efficiency, thinness, low cost, easy processing, flexibility, and response to environmental stimuli, a plethora of high-quality optical devices have been demonstrated. Here, a miniature planar telescope mediated by liquid crystal polymers is proposed to offer angle magnification independent of incident spatial location. It consists of two cascaded liquid crystal planar optical elements, each performing a predefined mathematical transformation. By this concept, planar optical elements are fabricated using a new exposure method and assembled into planar telescopes with different magnification factors. Within the incident field range, over 84.6% optical efficiency is achieved with small wavefront distortion. Such a miniature planar telescope shows the potential of cascaded liquid crystal planar optical elements for realizing functionalities that cannot be fulfilled by single optical elements, and enables lightweight, low loss, passive optical transmitters for widespread applications.

## Abstract (web summary)

A cascaded liquid crystal polymer-based miniature angle magnifier is demonstrated to enable high-efficiency, wide-range, and high-precision beam steering for light detection and ranging (LiDAR) applications.

## Graphical abstract

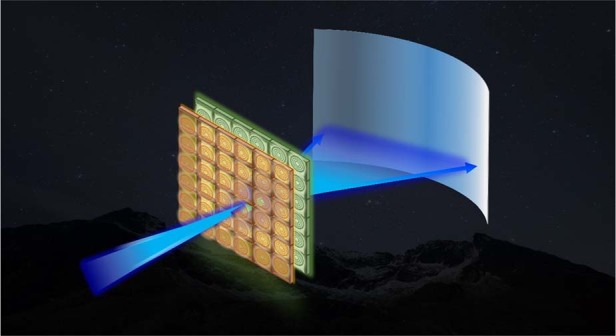

## Introduction

The telescope is one of the most important inventions in the history of optics, which can be traced back to the early 1600s^1^. It magnifies the incident angle of light by a certain ratio regardless of the incident position on the objective lens. With a large magnification factor and excellent optical quality, it enables the observation of distant objects, making it an indispensable tool in astronomy. In fact, such an operation principle is very useful for non-mechanical laser beam manipulating systems. Precisely positioning a laser beam has been proven to be pivotal for widespread applications including light detection and ranging (LiDAR)^2^, microscopy^3^, optical tweezers^4^, and laser micro-machining^5^. In addition, the recent developments of autonomous vehicles^6^, near-eye displays^7^, and high-precision 3D printing^8^ have aroused an urgent need for lightweight, compact, high-efficiency, high-precision, and/or large-angle beam steering technology, which is still missing. The most mature non-mechanical beam steering technology, termed optical phased array (OPA), offers quasi-continuous laser beam positioning but can maintain high efficiency only within a relatively small steering angle range (e.g., ±5°)^9–11^. Combined with dielectric metasurfaces, >35% efficiency has been realized for a large beam deflection angle of 11° at red wavelengths (~650 nm)^12^. To further enlarge the steering range while maintaining high efficiency, coupling an OPA with a telescope system is a viable approach. However, it is quite challenging to obtain a compact, low-cost, lightweight, and high-quality telescope module with conventional optics^13^.

Planar optical elements (POEs) based on patterned liquid crystals (LCs) have recently received extensive research interest^14–17^. Unlike dielectric metasurfaces which are usually fabricated by a sophisticated lithography process, LC planar optics, thanks to the self-assembly properties of LCs, can be simply created by directly coating LC monomers on a substrate (or filled in a cell) with predefined molecular alignment patterns^18^. Such patterns can be easily obtained through photoalignment techniques or others^19–25^, and the thickness of the alignment layer is usually in the order of 10 nanometers. Progresses have been made in delivering high-quality lenses^26–28^, gratings^29,30^, optical vortex processors^31–33^, etc. Using a chiral liquid crystal, even sub-wavelength grating pitches can be obtained with simple fabrication techniques^34,35^. Engineering of the operating spectral/angular bands in these optical devices has also been illustrated in both passive and active means. For example, polymerized multi-twist structures can be introduced to broaden the spectral/angular bandwidth as a passive means^29,30^. Meanwhile, active devices leverage the properties of LCs that can respond to external stimuli such as mechanical stress, electric fields, and light^14,36–39^. However, all the above-mentioned explorations are focused on functionalities that can be realized in a single-layer device. By transitioning from single layer to multilayer, more novel and distinct functionalities can be rationally designed^40,41^, while still maintaining the advantages of LC planar optics, such as high efficiency, thinness, low cost, lightweight, easy processing, and flexibility, etc.

Herein, we propose a cascaded LC planar optical element to achieve a miniature planar telescope for improving the performance of quasi-continuous laser beam steerers. The planar telescope enlarges the incident angle of light by a scalar factor, independent of the incident position. This unique feature has not yet been demonstrated with single-layer POEs. In experiments, different POEs with pre-designed phase profiles are fabricated using patterned LC polymers and assembled into miniature planar telescopes with different magnification factors. With the help of a planar telescope, the steering angle range can be enlarged greatly without losing too much power. Further characterization ensures the high quality of the output beam. Such a miniature planar telescope shows the potential of LC polymer-based cascaded POEs in achieving lightweight, low-power, cost-effective optical components, which hold great promise for practical applications.

## Results

### Angle magnification by a planar telescope

**Fig. 1:**
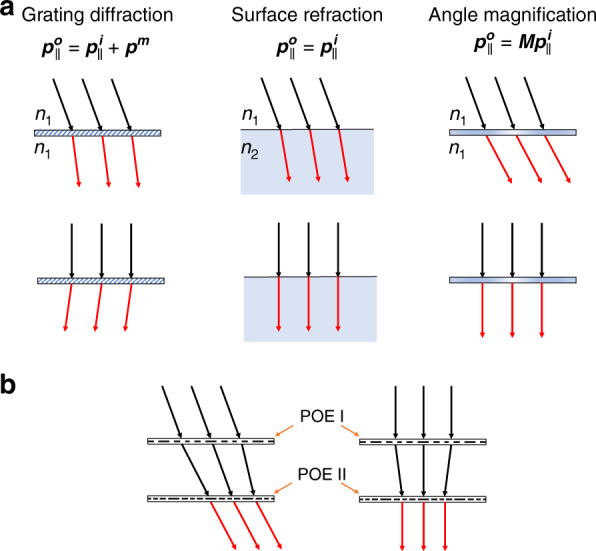
Angle magnification concept. **a** Schematic of light direction adjustment through grating diffraction, surface refraction, and angle magnification. In the first case, the grating adds a constant in-plane momentum component to the incident light independent of its incident angle. In the second case, upon surface refraction, the in-plane momentum component is conserved, but the output angle is scaled to the incident angle by Snell’s law. In the third case, for an ideal angle magnification, the device adds an in-plane momentum component to the incident light with a fixed scaling factor, and the input and output media are the same. **b** Illustration of a planar telescope consisting of two POEs for achieving angle magnification. Both POEs have spatially variant in-plane momentum. POE II compensates the spatially variant in-plane momentum of light added by POE I. Together, they work as a grating with adaptive periods according to the incident angle. ${\boldsymbol{p}}_\parallel ^{\boldsymbol{o}}$ in-plane momentum component of the output light, ${\boldsymbol{p}}_\parallel ^{\boldsymbol{i}}$ in-plane momentum component of the incident light, ${\boldsymbol{p}}^{\boldsymbol{m}}$ in-plane momentum component of the grating, ***M*** magnification factor, POE planar optical elements.

Figure 1a compares three different ways of controlling the light direction independent of the incident position, including grating diffraction, surface refraction, and angle magnification. For diffraction gratings, the grating imparts a fixed in-plane momentum component to the incident beam. Therefore, the output beam angle does not have a constant magnification to the incident angle. For surface refraction, the in-plane momentum is conserved at the boundary of two different media. In the paraxial approximation, the output beam can have a fixed magnification according to Snell’s law. However, in terms of actual application scenarios, the input and output media should be the same, and there will be no magnification. In comparison, for an ideal angle magnification, the incident beam angle should be scaled up with a fixed magnification factor (under paraxial approximation) and different input and output media are not required. In this case, the device adds different in-plane momentum components to beams with various input angles. This function can hardly be realized with only a single-layer POE.

A cascaded POE with a designed phase gradient is feasible to realize angle magnification, where the simplest case is stacking two POEs, as Fig. 1b depicts. For convenience, we assign certain in-plane momenta to POE I ($p_1(x_1)$) and POE II ($p_2(x_2)$), and call the separation distance between these two POEs as *d*. Since the LC polymer-based POE is thin (in the order of 1 μm), in the paraxial approximation, its response can be regarded as local. Then, it is easy to prove that if one of the POEs has a spatially invariant in-plane momentum ($p_i\left( {x_i} \right) = C$, where *C* is a constant), the cascaded device cannot have an angle magnification factor other than 1. Therefore, these two POEs require spatially variant in-plane momentum. In this manner, the minimum requirement demands two POEs with linearly varying in-plane momentum, $p_1\left( {x_1} \right) = c_1x_1$ and $p_2\left( {x_2} \right) = c_2x_2$, where *c*_*i*_ is the first-order constant. It can be shown that if the condition, $- \frac{d}{k} = \frac{1}{{c_1}} + \frac{1}{{c_2}}$ (*k*, the wavevector of light), is satisfied, the cascaded POE will perform an angle magnification function with a scaling factor of $M = - c_2/c_1$ (Supplementary Note 1). This is essentially the working principle of traditional telescopes with a thin-lens approximation. However, none of the traditional counterparts can provide low cost, lightweight, and a planar shape, which are highly desired for compact beam steerers.

To realize a planar telescope, at least two POEs with linearly varying in-plane momenta are required. In 2D space, this means lenses with a parabolic phase profile. Through ray-tracing simulations, the cascaded POEs with simple parabolic phase profiles are proven to perform the angle magnification function. However, as the incident angle increases, the quality of the output beam spot deteriorates slightly (Supplementary Fig. S1a). Although this slight degradation would not be critical for many applications, the off-angle performance can be further improved by incorporating higher-order phase terms. An example of optimized designs is shown in Supplementary Fig. S2. Compared to the design with parabolic phase profiles, its off-angle performance is greatly improved (Supplementary Fig. S1b).

### Liquid crystal planar optical elements

Attributed to the large birefringence and self-assembly nature, LCs are excellent materials to realize geometric phase (GP, also known as Pancharatnum–Berry phase) based POEs^14,15^. For transmissive-type POEs, GP is patterned by having circularly polarized input light transmitting through an LC layer with spatially varying anisotropy. The GP modulation can be predicted and designed by Jones matrix calculus. For an LC wave plate with a spatially varying director (local optical axis) distribution *φ*(*x,y*) in the *x–y* plane, the Jones matrix upon normal incidence can be described by^15^:

1 $$\begin{array}{lll}{\mathbf{J}} &=& {\mathbf{R}}( - \varphi )\left[ {\begin{array}{*{20}{c}} {e^{ - i\Gamma /2}} & 0 \\ 0 & {e^{i\Gamma /2}} \end{array}} \right]{\mathbf{R}}(\varphi ) \\&=& \cos \frac{\Gamma }{2}{\mathbf{I}} - i\sin \frac{\Gamma }{2}\left[ {\begin{array}{*{20}{c}} {\cos \left( {2\varphi } \right)} & {\sin \left( {2\varphi } \right)} \\ {\sin \left( {2\varphi } \right)} & { - \cos \left( {2\varphi } \right)} \end{array}} \right]\end{array}$$

where **R** is the rotation matrix, **I** is the identity matrix, and Γ is the LC phase retardation. The phase retardation is related to LC layer thickness (*t*), birefringence (Δ*n*), and incident light wavevector (*k*) as Γ = *t*Δ*nk*. With a circularly polarized input light ($${\mathbf{E}}_{\mathrm{i}} = \frac{1}{{\sqrt 2 }}\left[ {\begin{array}{*{20}{c}} 1 \\ { \pm i} \end{array}} \right]$$), the output light (${\mathbf{E}}_{\mathrm{o}}$) can be formulated as:

2 $${\mathbf{E}}_{\mathrm{o}} = {\mathbf{J}} \cdot {\mathbf{E}}_{\mathrm{i}} = \cos \frac{\Gamma }{2}{\mathbf{E}}_{\mathrm{i}} - \frac{i}{{\sqrt 2 }}\left[ {\begin{array}{*{20}{c}} 1 \\ { \mp i} \end{array}} \right]\sin \frac{\Gamma }{2}e^{ \pm i2\varphi }$$

Equation (2) has several implications: (1) The output has two orthogonal polarization states where the spin-flipped part is imposed of GP of ±2*φ*. A continuous phase change from 0 to 2π can be obtained by rotating the LC directors from 0° to 180°, and thus the wavefront of a circularly polarized input can be engineered by mapping the LC directors on the *x–y* plane. (2) The efficiency of the spin-flipped part depends on the retardation as $\sin ^2\left( {{{\Gamma }}/2} \right)$ such that a half-wave retardation results in maximum efficiency.

Taken a cascaded POE with parabolic phase profiles as an example, if a nominal focal length *f*_*i*_ is assigned to each POE, the first-order constant *c*_*i*_ is related to *f*_*i*_ as *c*_*i*_ = −*k/f*_*i*_, the distance between these two POE becomes $d = f_1 + f_2$, and the magnification factor turns out to be $M = - f_1/f_2$. To achieve a compact design with large magnification, the magnitude of *f*_1_ should be much larger than that of *f*_2_ while having an opposite sign. Meanwhile, the form factor should be on a microscale. Several methods have been realized to create LC alignment for microscale POEs, including direct laser writing^22^, nanoimprinting^24^, meta-mask projection^28^, and relayed polarization holography^42^. Among them, polarization holography is most cost-effective and creates a continuous phase profile. However, previous polarization holography can hardly be employed to create small *f*-number (*f*/#) microlenses due to the existence of a bulky beam splitter in front of the exposure plane. Here we propose a new method, namely counter-propagating wave polarization holography (see Supplementary Fig. S3 and “Materials and methods” section for details), to generate the desired LC alignment patterns. By this strategy, the two circularly polarized recording beams are incident on the exposure plane from opposite directions, which eliminates the issue of using a beam splitter in front of the exposure plane.

**Fig. 2:**
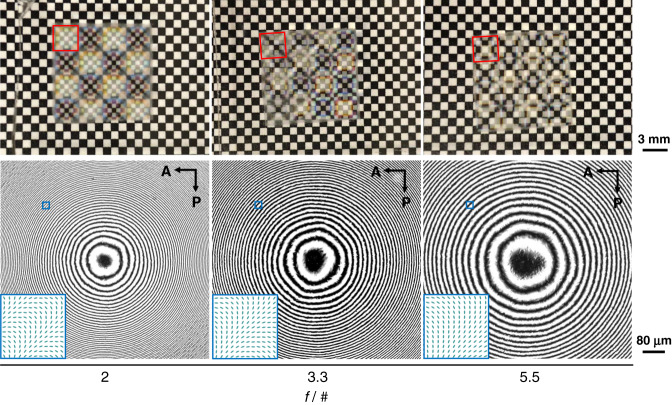
Characterization of fabricated polymerized LC POEs. Top row: Optical images of 4-by-4 arrays of fabricated *f*/2, *f*/3.3, *f*/5.5 POEs. The POEs are placed 6-mm away from the checkerboard background. The imaging effect of POEs can be clearly identified. The red squares highlight individual POEs. Bottom row: Polarized optical microscope images of the fabricated POEs with various *f*/#. The POEs are inserted in between two linear polarizers. The intensity modulation demonstrates spatially varying LC alignment. For a POE with a smaller *f*/#, such spatial variation of LC director distribution is more dramatic. The cyan squares highlight the LC director distribution of the selected area. A analyzer, P polarizer.

In experiments, three types of polymerized LC POEs were fabricated, with *f*/# of 5.5, 3.3, and 2.0, respectively. The top row of Fig. 2 shows the visual images of the POE arrays placed above a checkerboard. The size of each square POE is 3-by-3 mm^2^ and the distance between POE and checkerboard background is 6 mm. For the *f*/2 POE, the nominal focal lengths are ±6 mm for the two orthogonal circular polarization states. In this case, only the image of the −6-mm lens can be clearly identified. A similar imaging effect can be noticed for *f*/3.3 and *f*/5.5 POEs. The center view of the polarized optical microscope (POM) images for the POEs is manifested in the bottom row of Fig. 2. The fringes with gradually changing periods clearly illustrate the desired phase pattern. The inset (cyan squares) presents the LC director distribution in the selected area, which rotates continuously along the radial direction. For POEs with a larger *f*/#, the period of the fringes under POM is obviously larger. It is important to mention that for the polarization holography-based exposure method, the final phase pattern on the samples relies mostly on the shape and quality of the template.

### Beam steering performance of planar telescopes

**Fig. 3:**
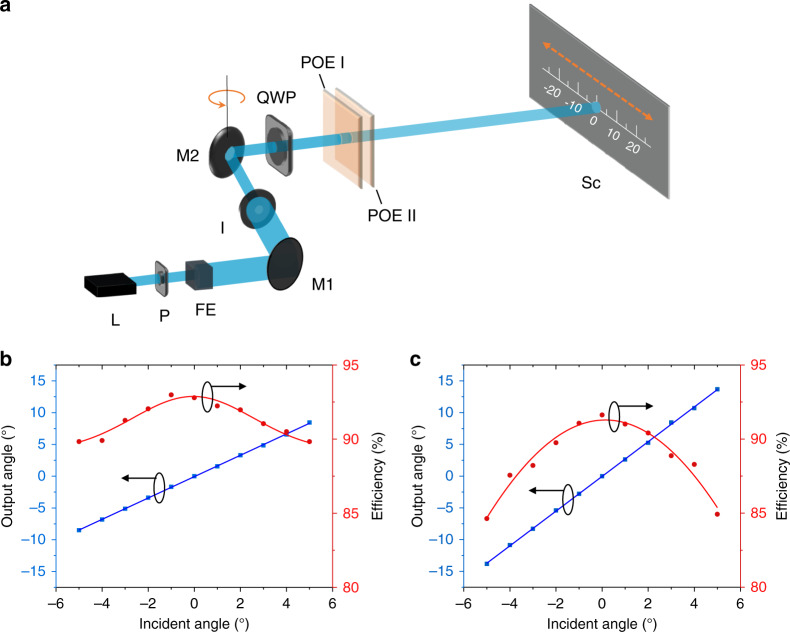
Miniature planar telescope efficiency and angle magnification. **a** Schematic of the measurement setup. A 488-nm circularly polarized laser beam is incident on the planar telescope composed of two POEs. A mirror (M2) is fixed on a rotational stage for the manipulation of incident angles. L laser, P polarizer, FE filtering and expansion, M1 mirror I, I iris, M2 mirror II, QWP quarter-wave plate, Sc screen. **b** Performance of module I which is composed of an *f*/5.5 POE I and an *f*/3.3 POE II. **c** Performance of module II which is composed of an *f*/5.5 POE I and an *f*/2 POE II. The measured data are presented by symbols and the solid lines are visual guides. The ovals and arrows highlight the *y* axis to which the data set corresponds. See the “Materials and methods” section for measurement details.

To evaluate the performance of proposed miniature planar telescopes, a measurement setup is established to mimic how they are employed in practical applications. As shown in Fig. 3a, a collimated, circularly polarized laser beam (*λ* = 488 nm) is incident on the cascaded POEs, and its incident angle is controlled by a rotatable mirror (M2). Two planar telescope modules based on the cascaded POEs are tested. Module I consists of an *f*/5.5 POE I and an *f*/3.3 POE II, while module II is composed of an *f*/5.5 POE I and an *f*/2 POE II. The distance between two POEs is controlled as ~6.6 mm for module I and ~10.5 mm for module II. The output angle and steering efficiency as a function of incident angles are recorded, as depicted in Fig. 3b, c, for modules I and II, respectively. By fitting the output angle and incident angle relationship with a linear function, module I shows a slope of 1.68, which agrees well with the designed magnification factor, 1.67. Such an agreement also holds for module II, whose measured slope is 2.73 and the designed slope is 2.75.

For both modules, the measured steering efficiency stays higher than 84.6% (module I: >89.8%; module II: >84.6%), which ensures low energy loss upon angle magnification within the incident field of view. Due to laser safety concerns, the low loss is a highly desirable feature in practical beam steering applications such as autonomous vehicles and near-to-eye displays. Note that for normal incidence, the efficiency does not reach 100%, which may be ascribed to the slight haze of the POEs and the slight deviation of the optimal operation wavelength. Per our measurement, the haze of *f*/5.5, *f*/3.3, and *f*/2 POEs is 1.7%, 2.2%, and 3.4%, respectively. The haze originates from several factors. For example, larger surface roughness of the LC polymer will result in a larger haze, and LC misalignment can also contribute. But fortunately, a better coating development can further improve the overall quality and bring even higher steering efficiency^26,29^. As a reference, commercial-quality inch-size LC geometric phase lenses with similar *f*/# can obtain haze as low as 1%. On the other hand, the optimal operation wavelength for these POEs is measured by placing the POEs in between two circular polarizers and recording their transmission spectra. As shown in Supplementary Fig. S4, the optimal wavelength for *f*/5.5, *f*/3.3, and *f*/2 POEs is 502, 510, and 492 nm, respectively. Such a slight deviation will contribute negligible efficiency drop (<1%) according to the $\sin ^2\left( {{{\Gamma }}/2} \right)$ law. For oblique incidence, efficiency drop can be observed for both modules and is more pronounced for module II. This can be understood by the incident angle dependency on the POE. As the POM images illustrate, the POEs show gradient local grating pitches, and the smallest grating pitch of *f*/2 POE (~2.0 μm, parabolic phase) is much smaller than that of *f*/3.3 (~3.2 μm, parabolic phase) POE. For a smaller grating pitch, the decreased first-order diffraction efficiency as a function of incident angles is more obvious, as numerically calculated in Supplementary Fig. S5. Nevertheless, this is not fundamentally limiting the device performance as multi-twist LC structures have already been demonstrated to compensate for the off-angle efficiency loss^30^.

Except for the energy loss caused by haze, operation wavelength deviation, and off-angle light leakage, the rest should be attributed to the wavefront aberration of POEs originating from the fabrication method we applied. Here, an off-the-shelf (unoptimized) aspherical lens is employed as the template in our interference exposure photoalignment patterning. To eliminate this wavefront aberration, a better template lens can be customized, or a different photo-patterning method such as direct laser writing or meta-mask projection can be implemented. Our ray-tracing simulation (Supplementary Fig. S1) proves that if the designed phase profiles are assigned to both POEs, the entire device can have almost diffraction-limited performance. As a result, this part of energy loss can be fully compensated by better engineering. If we take all the possible loss compensation methods into consideration, in the ideal case, the efficiency can achieve as high as 98%.

**Fig. 4:**
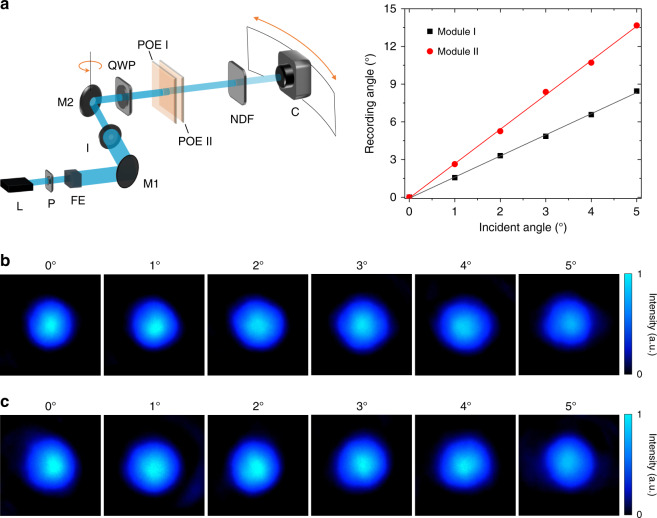
Angle-magnified beam shape characterization. **a** Left: Schematic of the measurement setup. A 488-nm circularly polarized laser beam is incident on the planar telescope composed of two POEs. A mirror (M2) is fixed on a rotational stage for controlling the incident angle. A camera coupled with a neutral density filter is applied to record the output laser beam spots and the location of the camera shifts according to the rotation angle of M2. Right: The recording angle of the camera as a function of the incident angle. L laser, P polarizer, FE filtering and expansion, M1 mirror I, I iris, M2 mirror II, QWP quarter-wave plate, NDF neutral density filter, C camera. **b** The recorded beam shape of module I as a function of incident angles. **c** The recorded beam shape of module II as a function of incident angles.

For some laser beam steering applications, maintaining the beam shape after steering is important. To record the output beam shape, a characterization setup is established, as depicted in Fig. 4a (left). The beam shape as a function of incident angle for both modules is captured, by adjusting the camera position with respect to the incident angle (Fig. 4a, right). The recorded beam shape is plotted in Fig. 4b, c, for modules I and II, respectively. Within the incident angle range, the output beam maintains a circular shape. More importantly, no optical vignetting is observed. This means, within the range of ±5° incidence on POE I, all the deflected light is directed to the POE II, and thus maximum efficiency is reached.

**Fig. 5:**
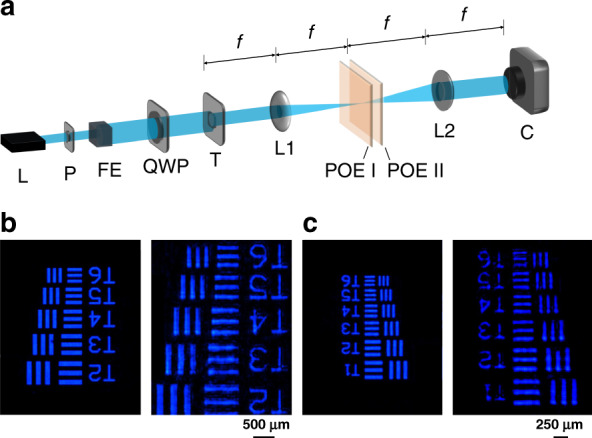
Miniature planar telescope wavefront characterization. **a** Schematic of the measurement setup. A 488-nm circularly polarized laser beam is incident on the 1951 USAF resolution test chart which is then imaged by a 4*f* system. A planar telescope module composed of an *f*/3.3 POE I and an *f*/2 POE II is placed on the 2*f* plane. Images with and without the planar telescope module are recorded. L laser, P polarizer, FE filtering and expansion, QWP quarter-wave plate, T test chart, L1 lens I, L2 lens II, C camera. The focal length of L1 and L2 is 15 cm. **b** Captured images of group 2 of resolution test chart without (left) and with (right) planar telescope. **c** Captured images of group 3 of resolution test chart without (left) and with (right) planar telescope.

To further investigate the wavefront variation upon angle magnification by the proposed miniature planar telescope, a 4*f* imaging system shown in Fig. 5a is applied to characterize the imaging properties. First, the images of groups 2 and 3 in the 1951 USAF resolution test chart without POEs are captured, as shown in the left part of Fig. 4b, c, respectively. Then, a planar telescope module consisting of an *f*/3.3 POE I and an *f*/2 POE II with ~4 mm distance is inserted into the 4*f* system. The center of the planar telescope is located on the 2*f* plane. The corresponding images are presented in the right part of Fig. 4b, c. As observed, the planar telescope enlarges the spatial frequency of light on the Fourier plane, resulting in magnified images on the image plane. Most of the details are well restored, except for slight ghosting. The ghost images may be ascribed to the slight haze of the POEs, which scatters light on the Fourier plane. With improved coating, a much better imaging performance can be expected.

## Discussion

Here two planar telescope modules with an incident angle range of ±5° and an operation wavelength of 488 nm have been demonstrated. For some beam steering applications, a longer operation wavelength is usually applied, e.g., near-infrared, mid-infrared, or even longer wavelengths^9,10^. In this system, the maximum output angle is limited by the minimum period of the POEs and the maximum diffraction angle can be estimated via diffraction equation. Note the minimum local grating pitch that polymerized LC POEs can offer has a weak correlation with the operation wavelength. In that sense, for a longer wavelength, the maximum output angle range and thus the magnification factor could be further enlarged. As an example, assuming a working wavelength of 905 nm, a minimum local grating pitch of 2 μm, an incident field range of ±5°, and no optical vignetting, then a maximum output angle range of ±26.9° and thus a magnification factor of 5.4 can be expected. Similarly, for *λ* = 1550 nm, the steering range can be easily expanded to >±30°. In the proof-of-principle demonstration, 1D magnification has been shown. However, since the device is rotationally symmetric (lens profile), it should be compatible with 2D beam steering. For instance, if the planar telescope is coupled to a 2D OPA or two 1D OPAs, it can enlarge the steering range in both dimensions. In comparison with other approaches (Supplementary Table 2), the proposed approach only adds a compact, passive optical device to an existing OPA, which is highly promising to achieve wide-angle, ultra-high efficiency (98% in the ideal case), compact beam steerers even for short wavelengths.

Due to the dispersion of diffractive optics, the current device is not broadband. For laser beam steering applications targeting a specific operation wavelength, it works well. If the application targets a broader spectral range, then the device needs further optimization, for example, changing the single POE to an achromatic doublet, or modifying the design by adding more POEs to compensate for the chromatic aberration.

The planar telescope presented in this work has a millimeter scale and thickness. To further reduce thickness, submicron scale POEs can be a good choice due to the device scalability. If scaled-down, a 2D array of POEs may be applied to extend the aperture size of the planar telescope. However, even with seamless tiling of the POEs, the output wavefront will be modulated by the array in this case. For beam steering applications without strict requirements on the output wavefront, this is still a quite promising route. Besides working as magnifiers, the planar telescope can also scale down the incident angles by properly choosing the combination of POEs. Through scaling down the incident angles, the steering range is decreased but the steering precision is improved. High-precision laser beam steering is particularly useful for applications with a requirement on ultra-high steering resolution over a narrow steering range, such as fine tracking for space communication.

In conclusion, lightweight, low-cost, miniature planar telescopes enabled by polymerized LC POEs are demonstrated and evaluated. The planar telescope shows high efficiency, engineerable magnification factor, and small wavefront distortion, which is highly promising in practical applications requiring advanced laser beam steering technology. Moreover, the planar telescope proves the potential of cascaded POEs based on LC polymers, and should enlighten more novel and elaborated optical designs for practical uses.

## Materials and methods

### Simulation and optimization

The ray-tracing simulations are performed using commercial optical design software (Zemax OpticStudio). The simulation is based on a 488-nm monochromatic light source with a 2-mm beam diameter. The diameter of the POEs is 3 mm, and the distance between two POEs is 10.5 mm. The optimized design is obtained by minimizing the angular spread of the output beam while maintaining a correct angle magnification factor. The phase profiles (*ϕ*) of the POEs are defined as:

3 $$\phi \left( r \right) = \mathop {\sum}\limits_{i = 1}^3 {a_i\left( {\frac{r}{R}} \right)^{2i}}$$

where *r* is the radial coordinate, *R* = 1.5 mm is the radius of the POE, *a*_i_ is the coefficient. The angular spot diagrams and root-mean-square (RMS) wavefront error for both designs are demonstrated in Supplementary Fig. S1a, b, respectively. The optimized phase profiles of the POEs are manifested in Supplementary Fig. S2, and the values of *a*_i_ for both designs are listed in Supplementary Table 1. The rigorous coupled-wave analysis solver is established in MATLAB. The guideline of constructing the solver can be found in a prior art^43^.

### Counter-propagating wave polarization holography

The optical setup of counter-propagating wave polarization holography is schematically plotted in Supplementary Fig. S3. An OBIS 488-nm LS 60-mW laser (Coherent) is employed as the exposure source. After filtering and expansion, the linearly polarized laser beam is split into two arms by a non-polarizing beam splitter. Two quarter-wave plates are placed on the two arms to convert the linear polarization to circular polarizations. After being redirected by the mirrors, the two arms are counter-propagating, and the circular polarization has the same handedness with respect to their own propagating direction. The mathematical principle of polarization field generation is similar to the standing wave polarization holography^44,45^. However, the standing wave method previously demonstrated is not compatible with exposing small *f*/#, small size lenses. In our setup, a template lens (L1) with a small *f*/# is placed on one arm to create the desired phase pattern. Another auxiliary lens (L2) with a large *f*/# is placed on the other arm to ensure that the two arms have nearly the same irradiance on the exposure plane. In experiments, the template lens is a commercial aspheric lens with *f*/1 (Edmund Optics) and the *f*/# of the auxiliary lens is ~20. To generate LC POE patterns with different *f*/#, the exposure plane and the auxiliary lens position are adjusted simultaneously. Two square photomasks are placed close to the exposure plane to ensure a square-shaped pattern. The array is exposed by a step-and-flash strategy.

The major advantage of this exposure method is its compatibility with exposing small *f*/# (such as *f*/2 or even smaller), small size (millimeter-scale or even smaller) lens patterns. Also, interference exposure methods provide a continuous pattern. There are some exposure methods developed by different groups, such as projection methods^25,46^, direct laser writing^22^, nanoimprinting^24^, meta-mask projection^28^, and relayed polarization holography^42^. The projection methods based on a digital micromirror device (DMD) or a spatial light modulator (SLM) suffer from pixelization effect and limited exposure area. The pixel size of a high-end DMD or SLM is usually ~4 μm (most low-cost devices have a pixel size larger than 10 μm). To fabricate a small *f*/# lens, the minimum grating pitch can be as small as 2 μm. Even with careful magnification control during projection exposure, it is still quite challenging to obtain a continuous pattern with high quality. For other methods such as direct laser writing and meta-mask projection, small *f*/# microlenses can be achievable. However, they require much advanced laser systems or nano-fabrication facilities. For nanoimprinting and relayed polarization holography, they can achieve microlenses, but only a relatively large *f*/# (~*f*/10) has been demonstrated. Therefore, here we customized the polarization holography method to make it compatible with exposing small *f*/#, small size patterns. For traditional holographic methods, usually, the two arms are combined with a beam splitter in front of the sample plane. This beam splitter is bulky and can add spherical aberration to the recording field. To record a small *f*/# lens, the distance between the template optics and the sample is so small (in our work, the shortest distance is ~1.2 cm) that it is quite unlikely to obtain such patterns with a beam splitter sitting in between.

### Polymerized liquid crystal planar optical element fabrication

First a thin film of the photoalignment material, 0.4 wt% Brilliant Yellow (BY, from Tokyo Chemistry Industry) dissolved in dimethylformamide (DMF) solvent, is spin-coated onto cleaned glass substrates with 500 rpm for 5 s and 3000 rpm for 30 s. Then, the substrate coated with BY is mounted on the exposure plane in the counter-propagating wave polarization holography setup for exposure, with a dosage of ∼1.2 J/cm^2^ (4 min with ∼5 mW/cm^2^ irradiance). After exposure, a reactive mesogen solution, consisting of 97 wt% reactive mesogen RM257 (from LC Matter) and 3 wt% photo-initiator Irgacure 651 (from BASF) dissolved in toluene with a weight ratio of 1:3, is spin-coated onto the substrates with 2000 rpm for 30 s. As the final step, the samples are cured by UV light (365 nm) for 5 min with ∼10 mW/cm^2^ irradiance.

### Polymerized liquid crystal planar optical element characterization

The POM images of POEs are captured by an Infinity 2-2 camera coupled with an optical microscope (OLYMPUS BX51). To estimate the haze of the samples at 488 nm, a collimated, circularly polarized 488-nm laser beam is incident on the samples and an iris is placed close to the sample to filter out the scattered light, letting only the first and zeroth orders pass through. A power meter (PM100D coupled with S130C, Thorlabs) is employed to record the transmitted power. The transmittance is normalized to the case where instead of the sample, a cleaned glass is placed on the optical path. Then, the haze is estimated as the power loss. Transmission spectra of samples are measured by a setup shown in Supplementary Fig. S4. The POE sample is placed between two circular polarizers with the same handedness. Halogen white light is applied as the light source and a fiber spectrometer (HR4000CG-UV-NIR, Ocean Optics) is used to record the transmission spectra.

### Miniature planar telescope characterization

The planar telescopes are characterized using the setups schematically shown in Figs. 3a, 4a, and 5a. In the efficiency and angle magnification measurements, the 488-nm laser is firstly expanded to a diameter of ~25 mm. Then, an iris is used to truncate the beam diameter to 2 mm with a uniform irradiance distribution. A mirror (M2) is mounted on a two-axis translation stage, which is mounted on a rotational stage. The POEs are aligned in the case of normal incidence, by assuring that the output beam is at a normal angle and has a minimum divergence. Through controlling the mirror (M2), the incident angle can be adjusted, and the output spot position can be read on the screen. The output angle is then calculated based on trigonometry. To measure the steering efficiency, a power meter is applied to record the power of the deflected beams. Note that the efficiency is normalized to the case where instead of the planar telescope module, two cleaned glasses are placed in the optical path. Therefore, efficiency is defined as the power of the deflected beam (with the planar telescope) over the power of the undeflected beam (with two cleaned glasses). In the beam shape characterizations, the optical setup is similar to the setup of angle magnification measurements. Instead of using a screen, a CMOS camera (EO5012 LE, Edmund Optics) coupled with a neutral density filter is applied to record the beam shape. In the wavefront characterization, the same 488-nm laser is applied as the light source. A 4*f* system with *f* = 15 cm is established and the CMOS camera is placed on the imaging plane to record the images.

## Supplementary information

Supplementary Information

## Data Availability

All data needed to evaluate the conclusions in the paper are present in the paper. Additional data related to this paper may be requested from the authors.
