# Supplementary material for: Miniature planar telescopes for efficient, wide-angle, high-precision beam steering
Source: Light Sci Appl. 2021 Jun 28;10:134. doi: 10.1038/s41377-021-00576-9 (PMC8239018; doi:10.1038/s41377-021-00576-9)
Supplement: Supplementary file 1 — Supplementary Information [file 41377_2021_576_MOESM1_ESM.docx]

**Supplementary information for**

**Miniature planar telescopes for efficient, wide-range, high-precision beam steering**

Ziqian He, Kun Yin, and Shin-Tson Wu

*College of Optics and Photonics, University of Central Florida, Orlando, FL 32816, USA*

*Correspondence: Shin-Tson Wu (email: swu@creol.ucf.edu)*

**Supplementary Note 1 | Theory of optical angle magnification**

Parameter definition: operation wavelength, *λ*; wave vector, *k*; input and output media, air; incident angle on POE I, $\theta_{1}$; incident angle on POE II, $\theta_{2}$; output angle, $\theta_{o}$; spatial coordinate on POE I, $x_{1}$; spatial coordinate on POE II, $x_{2}$; in-plane momentum of POE I, $p_{1}\left( x_{1} \right)=c_{1}x_{1}$; in-plane momentum of POE II, $p_{2}\left( x_{2} \right)=c_{2}x_{2}$; distance between POE I and POE II, *d*. Paraxial approximation is applied in derivation.

Light incident on POE I has an in-plane momentum of ${k\theta}_{1}$. After POE I, the in-plane momentum of light becomes ${k\theta}_{1}+c_{1}x_{1}$. After propagating by a distance, *d*, it will be incident on POE II at position $x_{2}={d(\theta}_{1}+\frac{c_{1}x_{1}}{k})+x_{1}.$ After POE II, the in-plane momentum of light becomes ${k\theta}_{1}+c_{1}x_{1}+c_{2}\left( {{d\theta}_{1}+\frac{dc_{1}x_{1}}{k}+x}_{1} \right).$ For optical angle magnification, the in-plane momentum of output light should be magnified by *M*, comparing to that of incident light. Therefore, ${k\theta}_{1}+c_{1}x_{1}+c_{2}\left( {{d\theta}_{1}+\frac{dc_{1}x_{1}}{k}+x}_{1} \right)=Mk\theta_{1}.$ After rearrangement, it can be described as $(k+c_{2}{d)\theta}_{1}+(c_{1}+c_{2}+\frac{dc_{1}c_{2}}{k})x_{1}=Mk\theta_{1}.$ This relationship should be independent of spatial position, $x_{1}$. Thus, $c_{1}+c_{2}+\frac{dc_{1}c_{2}}{k}=0$, and after rearrangement, it can be stated as $-\frac{d}{k}=\frac{1}{c_{1}}+\frac{1}{c_{2}}.$ If this condition holds, the magnification factor *M* can be presented as $M=1+c_{2}d/k=-c_{2}/c_{1}$.


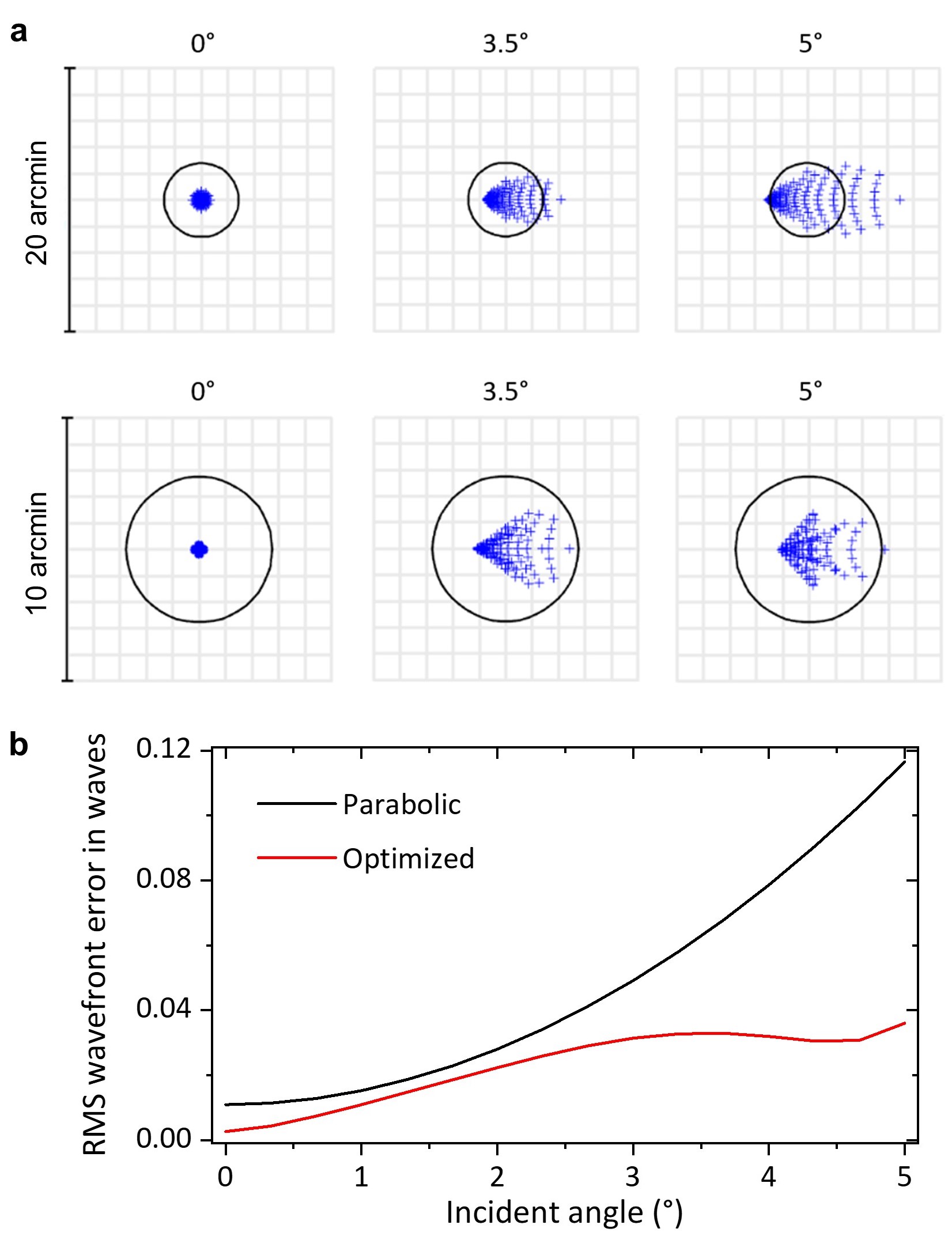


**Supplementary Figure S1 | Simulated performance comparison of two planar telescope designs.** (**a**) Angular spot diagrams of the two designs as a function of incident angles (0°, 3.5°, 5°). Top row: Parabolic phase; Bottom row: Optimized phase. The black circles demote Airy disks. (**b**) Root mean square (RMS) wavefront error of the transmitted light as a function of incident angles.


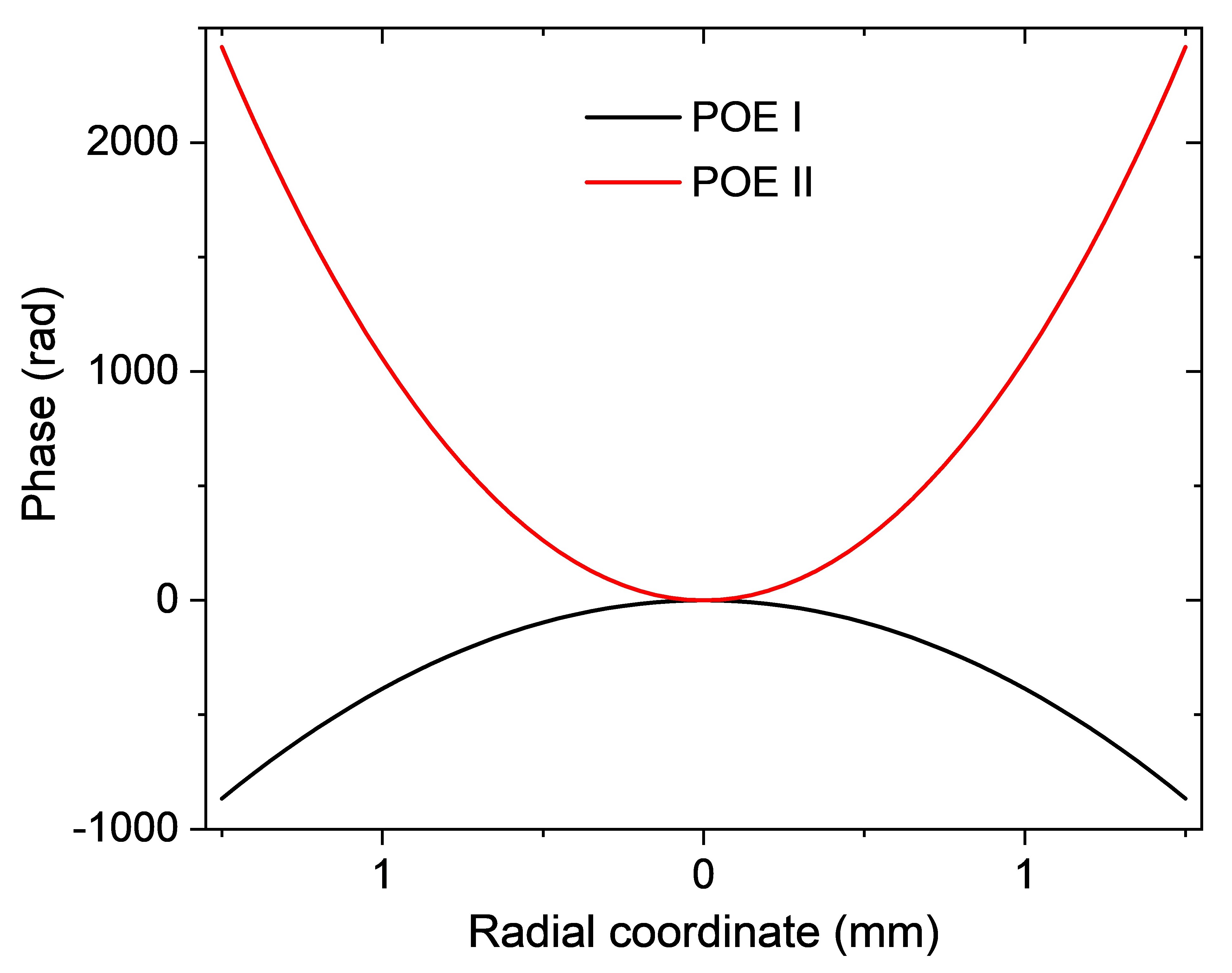


**Supplementary Figure S2 | Optimized phase profiles of the planar optical elements.** Optimized phase profiles of two planar optical elements consisting of the planar telescope with a magnification factor of 2.83.


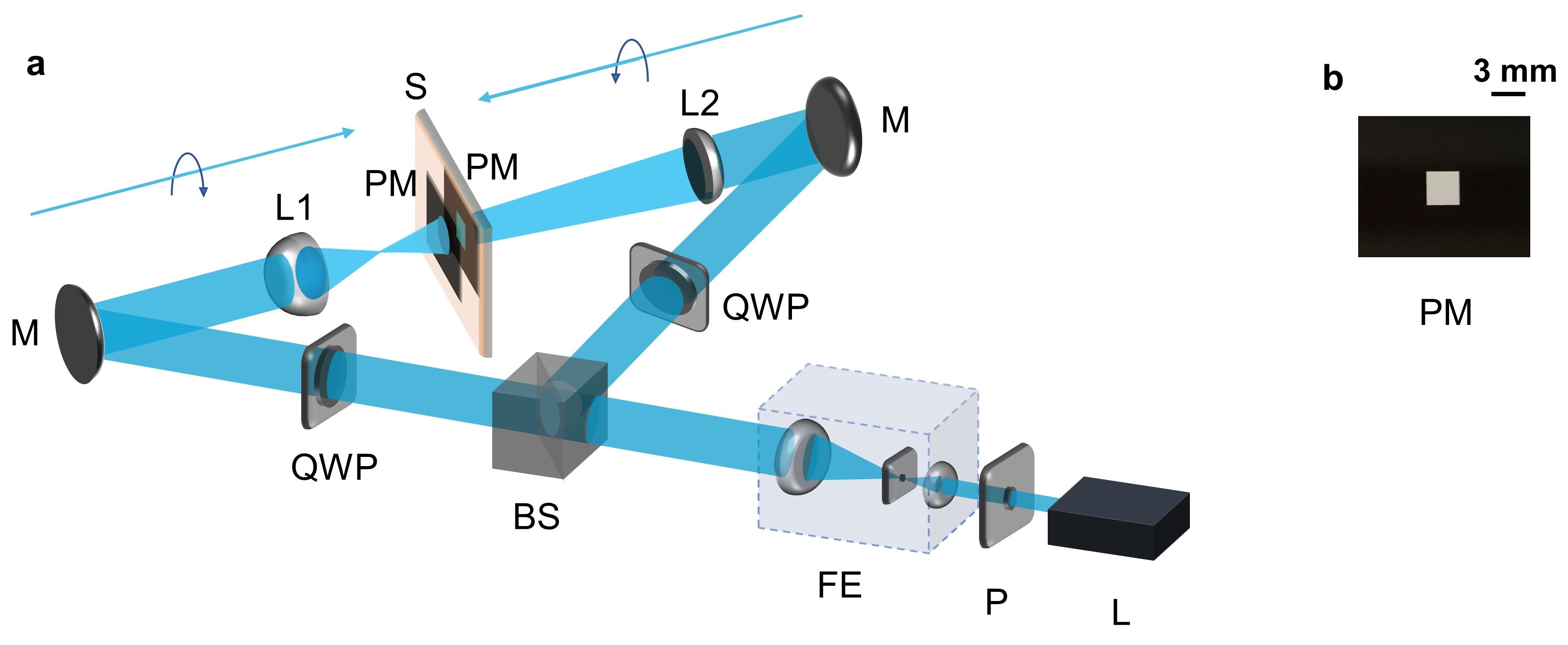


**Supplementary Figure S3 | Counter-propagating wave polarization holography.** (**a**) Schematic of the polarization holography exposure setup. A 488-nm laser is split into two arms. Both arms have the same handedness of circular polarization with respect to the propagating direction. L1 is a template aspherical lens with small *f*/#, and L2 is a lens with a focal length of 1 m. The purpose of L2 is to adjust the irradiance of light so that when the two arms interfere at the sample plane, they have nearly equal irradiance. P, polarizer; FE, filtering and expansion; BS, beam splitter; QWP, quarter wave plate; M, mirror; L1, template lens; L2, auxiliary lens; PM, photo mask; S, sample. (**b**) Image of the photomask with a dimension of 3-by-3 mm^2^.


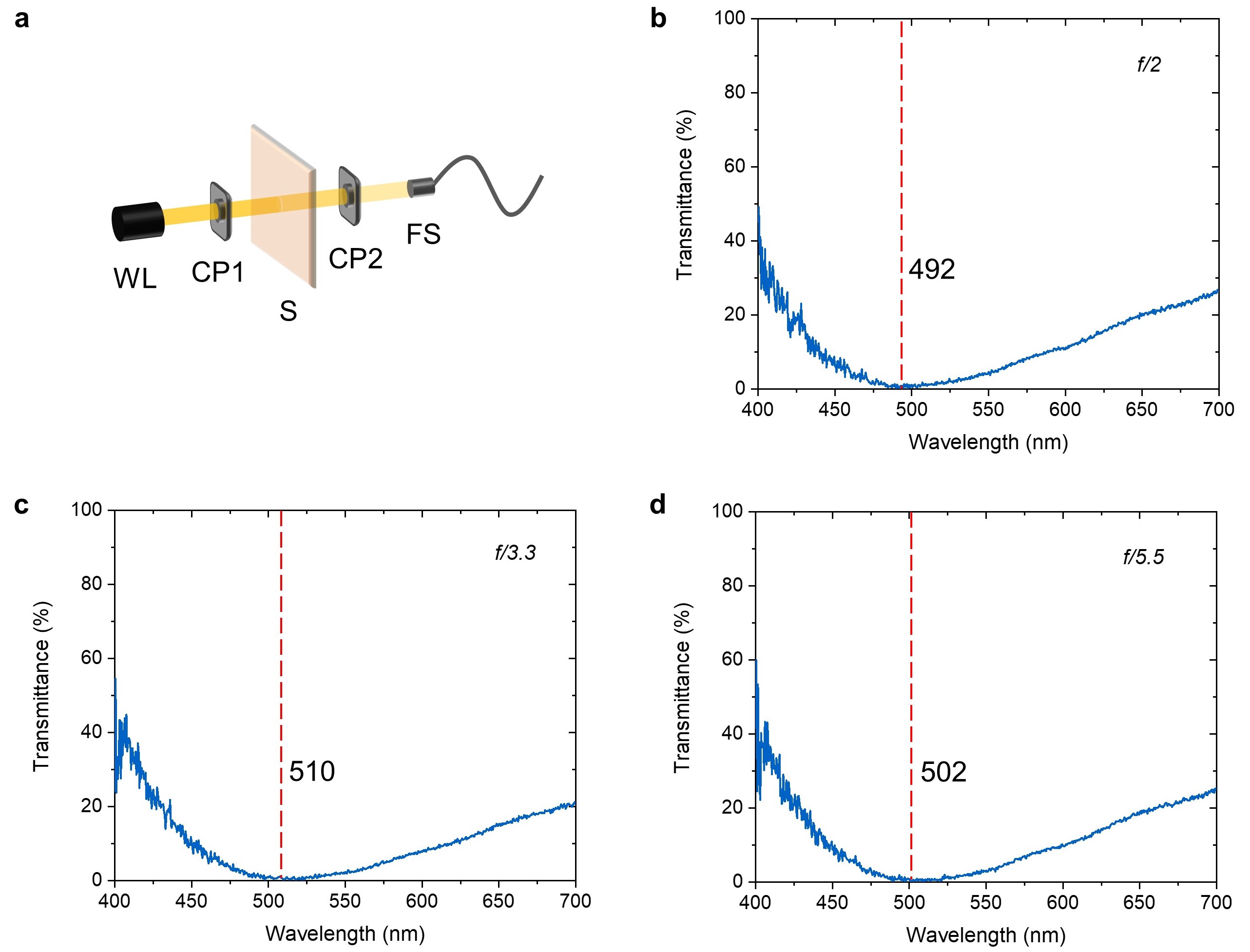


**Supplementary Figure S4 | Operation wavelength characterization.** (**a**) Schematic of the operation wavelength measurement setup. The POE sample is placed in between two circular polarizers with the same handedness. The light source is a white halogen light and a fiber spectrometer is utilized as the receiver. WL, white light source; CP, circular polarizer; S, sample; FS, fiber spectrometer. (**b**) Measured transmission spectrum of an *f*/2 POE. (**c**) Measured transmission spectrum of an *f*/3.3 POE. (**b**) Measured transmission spectrum of an *f*/5.5 POE.


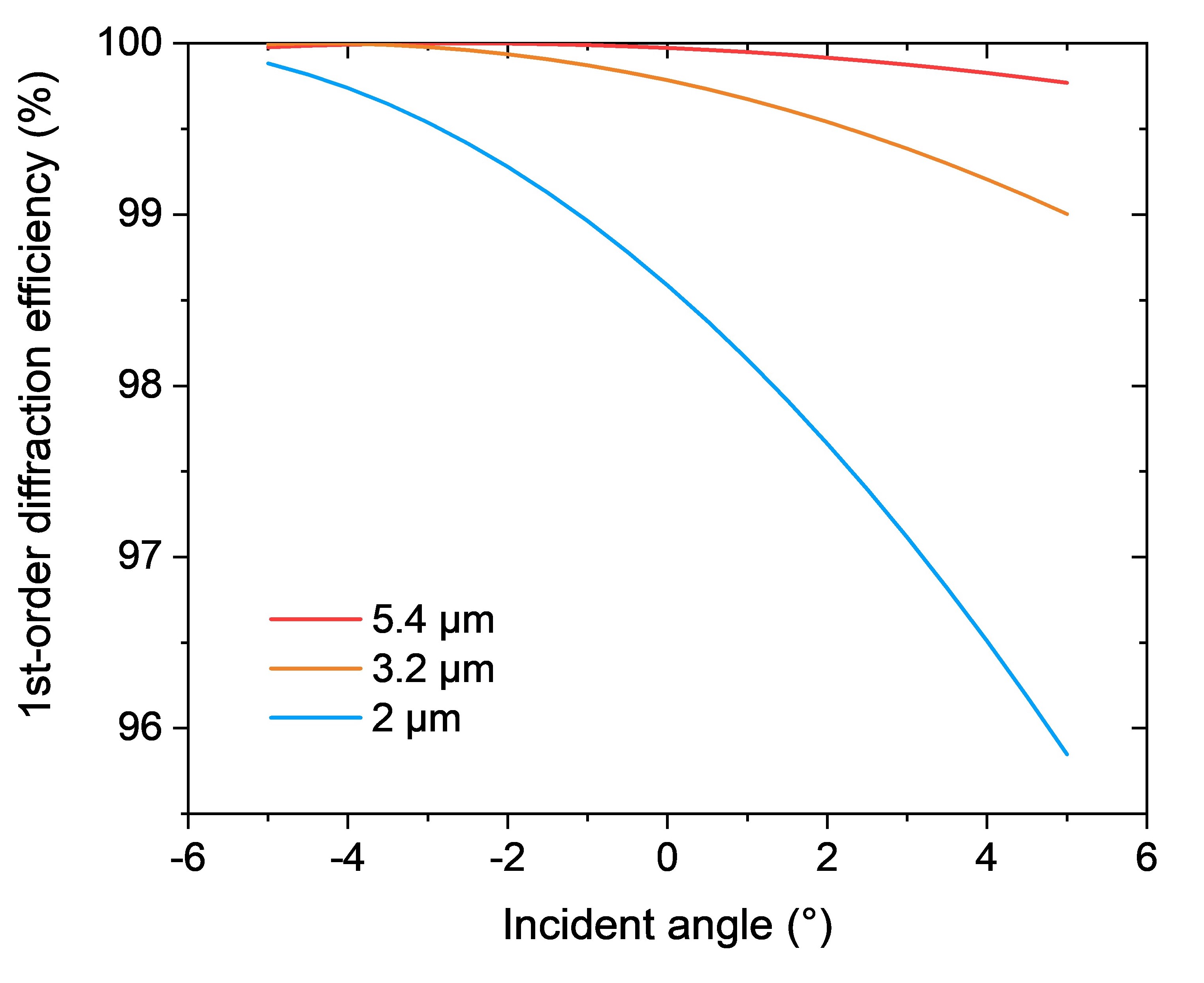


**Supplementary Figure S5 | Angle dependency on non-twist LC POEs.** Calculated incident angle-dependent first-order diffraction efficiency of non-twist LC POEs with a grating period of 2 μm, 3.3 μm, 5.4 μm, which corresponds to the minimum local grating period of *f*/2, *f*/3.3, *f*/5.5 POEs with a parabolic phase profile, respectively. The calculation is performed using a home-made rigorous-coupled wave analysis solver.

**Supplementary Table 1 | Phase profile coefficients for different designs**

|  | Parabolic | |  | Optimized | |
| --- | --- | --- | --- | --- | --- |
|  | POE I | POE II |  | POE I | POE II |
| *a*_1_ | -877.87 | 2414.13 |  | -870.58 | 2354.94 |
| *a_2_* | - | - |  | 2.75 | 40.98 |
| *a*_3_ | - | - |  | -1.96 | 22.15 |

**Supplementary Table 2 | Comparison among different LC-based beam steerers.** LC: liquid crystal; OPA: optical phased array; MS: metasurfaces; PG: polarization grating; WG: waveguide; PVG: polarization volume grating.

| Device | λ (nm) | Range (°) | Efficiency | Continuity | Compactness | Voltage |
| --- | --- | --- | --- | --- | --- | --- |
| LC-OPA [1] | 1550 | ±3.1^a^ | ~90% | Quasi-cont. | Compact | <6.5 V |
| LC-MS [2] | 650 | ±11 | >35% | Quasi-cont. | Compact | <8 V |
| PG Stack [3] | 1550 | ±22 | >94% | 27 stages | Medium | <10 V |
| PG Stack [4] | 1550 | ±32 | >93% | 8 stages | Medium | <10 V |
| LC WG [5] | 1550 | 50$\times$15 | >50% | Cont. | Bulky | <100 V |
| PVG [6] | 532 | 43.5~55 | >90% | Cont. | Compact | -^b^ |
| This work | 488 | ±14 (2D) | >85% | Quasi-cont. | Compact | Passive |
| This work^c^ | 905 | ±27 (2D) | >95% | Quasi-cont. | Compact | Passive |

^a^A larger range of ±7° is available without efficiency data. ^b^The mechanism is mechanical stretching such that the applied voltage depends on the stretcher. ^c^This is a projected result based on the existing fabrication techniques and optical designs.

**References**

1. Linnenberger, A., Serati, S., & Stockley, J. Advances in optical phased array technology. *Proc. SPIE* **6304**, 63040T (2006).
2. Li, S. Q. *et al.* Phase-only transmissive spatial light modulator based on tunable dielectric metasurface. *Science* **364**, 1087-1090 (2019).
3. Kim, J. *et al.* Wide-angle, nonmechanical beam steering with high throughput utilizing polarization gratings. *Appl. Opt.* **50**, 2636–2639 (2011).
4. Kim. J., Miskiewicz, M. N. & Escuti, M. J. Nonmechanical Laser Beam Steering Based on Polymer Polarization Gratings: Design Optimization and Demonstration. *J. Light. Technol.* **33**, 2068–2077 (2015).
5. Davis, S. R. *et al.* Liquid crystal clad waveguide laser scanner and waveguide amplifier for LADAR and sensing applications. *Proc. IEEE* **9365**, 93650N (2015).
6. Yin, K. *et al.* Stretchable, flexible, rollable, and adherable polarization volume grating film. *Opt. Express* **27**, 5814–5823 (2019).
